# New York State, New York City, New Jersey, Puerto Rico, and the US Virgin Islands' Health Department Experiences Promoting Health Equity During the Initial COVID-19 Omicron Variant Period, 2021-2022

**DOI:** 10.1089/hs.2023.0001

**Published:** 2023-09-27

**Authors:** Heidi Cox, Yonathan Gebru, Libby Horter, Francisco S. Palomeque, Kristopher Myers, Daniel Stowell, Torian Easterling, Nayeli Salazar de Noguera, Amanda Medina-Forrester, Josely Bravo, Siomara Pérez, Jaikiz Chaparro, Lisa La Place Ekpo, Hannah Cranford, Scott Santibañez, Diana Valencia

**Affiliations:** Heidi Cox, MPH, is a Public Health Analyst; in the Division of Preparedness and Emerging Infections, National Center for Emerging and Zoonotic Infectious Diseases, US Centers for Disease Control and Prevention (CDC), Atlanta, GA.; Yonathan Gebru, MPH, is a Public Health Advisor; in the Division of Preparedness and Emerging Infections, National Center for Emerging and Zoonotic Infectious Diseases, US Centers for Disease Control and Prevention (CDC), Atlanta, GA.; Libby Horter, MPH, is a Data Manager; in the Division of Preparedness and Emerging Infections, National Center for Emerging and Zoonotic Infectious Diseases, US Centers for Disease Control and Prevention (CDC), Atlanta, GA.; Francisco S. Palomeque, MPH, is a Health Scientist, Division of State and Local Readiness, National Center for Emerging and Zoonotic Infectious Diseases, CDC, Atlanta, GA.; Kristopher Myers, PhD, was a Consultant Data Manager, State, Tribal, Local, and Territorial Support Task Force, CDC, Atlanta, GA. He is currently a Data Manager, Goldbelt, C6, LLC, Chesapeake, VA.; Daniel Stowell, MPH, is a Public Health Analyst, Center for Global Health, CDC, Atlanta, GA.; Torian Easterling, MD, PhD, is First Deputy Commissioner and Chief Equity Officer, New York City Department of Health and Mental Hygiene, New York, NY.; Nayeli Salazar de Noguera, PhD, is a Program Management Officer, the New Jersey Department of Health, Trenton, NJ.; Amanda Medina-Forrester, MPH, is Executive Director of Office of Minority and Multicultural Health; the New Jersey Department of Health, Trenton, NJ.; Josely Bravo, MPH, is a COVID-19 Vaccine Equity Official; the Puerto Rico Department of Health, San Juan, PR.; Siomara Pérez, DrPH, is a Project Manager; the Puerto Rico Department of Health, San Juan, PR.; Jaikis Chaparro, MSW, is Director of Health Equity Program; the Puerto Rico Department of Health, San Juan, PR.; Lisa La Place Ekpo, DrPH, is Epidemiologists, US Virgin Islands Department of Health, Saint Thomas, USVI.; Hannah Cranford, MPH, is Epidemiologists, US Virgin Islands Department of Health, Saint Thomas, USVI.; Scott Santibañez, MD, MPHTM, is Chief Medical Officer and Associate Director for Science; in the Division of Preparedness and Emerging Infections, National Center for Emerging and Zoonotic Infectious Diseases, US Centers for Disease Control and Prevention (CDC), Atlanta, GA.; Diana Valencia, MS, is a Health Scientist; in the Division of Preparedness and Emerging Infections, National Center for Emerging and Zoonotic Infectious Diseases, US Centers for Disease Control and Prevention (CDC), Atlanta, GA.

**Keywords:** COVID-19, Health equity, Public health preparedness/response, Epidemic management/response, Partnerships

## Abstract

In this case study, we aim to understand how health departments in 5 US jurisdictions addressed health inequities and implemented strategies to reach populations disproportionately affected by COVID-19 during the initial Omicron variant period. We used qualitative methods to examine health department experiences during the initial Omicron surge, from November 2021 to April 2022, assessing successful interventions, barriers, and lessons learned from efforts to promote health equity. Our findings indicate that government leadership supported prioritizing health equity from the beginning of the pandemic, seeing it as a need and vital part of the response framework. All jurisdictions acknowledged the historical trauma and distrust of the government. Health departments found that collaborating and communicating with trusted community leaders helped mitigate public distrust. Having partnerships, resources, and infrastructure in place before the pandemic facilitated the establishment of equity-focused COVID-19 response activities. Finally, misinformation about COVID-19 was a challenge for all jurisdictions. Addressing the needs of diverse populations involves community-informed decisionmaking, diversity of thought, and delivery measures that are tailored to the community. It is imperative to expand efforts to reduce and eliminate health inequities to ensure that individuals and communities recover equitably from the effects of COVID-19.

## Introduction

Health equity is achieved when all members of society experience a fair and just opportunity to be as healthy as possible, with no hindrances based on their social status or circumstances.^1-3^ Promoting health equity requires continuous commitment to valuing all people and providing resources according to needs.^2^

The COVID-19 pandemic has challenged the capacity of public health agencies to advance health equity. Populations that have historically experienced discrimination and health inequities continue to be disproportionately impacted by the pandemic.^4-7^ Many health departments (HDs) across the United States have built upon decades of response best practices, and leveraged prior community engagement and partnerships, to attempt to reach historically disadvantaged populations experiencing the most severe COVID-19 outcomes.^7^ These efforts were complicated, in part, by the emergence of the Omicron SARS-CoV-2 variant of concern, first reported in the United States on December 1, 2021.^8-10^

In this case study, we describe how HDs in 5 US Department of Health and Human Services (HHS) Region 2 jurisdictions—New York State, New York City, New Jersey, Puerto Rico, and the US Virgin Islands—addressed health inequities and implemented strategies to reach populations disproportionately affected by COVID-19 during the initial Omicron variant period.

## Background

Over the past 2 decades, public health agencies have developed strategies to reach populations disproportionately affected by COVID-19 based on lessons learned from past public health emergencies.^4^ The December 2006 Pandemic and All-Hazards Preparedness Act^11^ required HHS to ensure the integration of planning for at-risk populations into emergency response policy and programs.^12^ In response, the US Centers for Disease Control and Prevention (CDC); federal, state, tribal, local, and territorial agencies; community-based organizations; faith-based organizations; and other partnership organizations engaged in efforts to promote health equity.^13^ CDC developed the *Public Health Workbook to Define, Locate, and Reach Special, Vulnerable, and At-Risk Populations in an Emergency*^14^ and worked with the Association of State and Territorial Health Officials and the National Association of County and City Health Officials to produce *At-Risk Populations and Pandemic Influenza: Planning Guidance for State, Territorial, Tribal, and Local Health Departments*.^15^ These early efforts laid the groundwork for similar efforts during the COVID-19 pandemic.^13^

Disparities in COVID-19 prevalence and mortality estimates were observed across jurisdictions and racial and ethnic populations.^16-21^ When the COVID-19 pandemic began, testing, physical distancing, case investigation, contact tracing, isolation, and medical care were critical interventions implemented to reduce transmission.^22^ As the pandemic progressed, COVID-19 vaccines were considered the most effective intervention to ending the pandemic.^23^ Historically, underserved communities have experienced lower adoption of interventions.^21^ This has been a challenge to HDs in their efforts to advance health equity.

Many underlying inequities experienced by populations disproportionately affected by COVID-19 are longstanding, such as a lack of linguistically and culturally accessible resources.^24^ Strategies to address these inequities involve the commitment of time and resources by HDs to develop community-driven approaches. Engaging people impacted by the circumstances of where they live in their own environment is critical to affecting health-associated behaviors and risks.^25^ Community-driven approaches rooted in partnerships with trusted community members to promote capacity building, power sharing, colearning, and cocreation provide a broader social justice perspective than more general approaches.^26^

## Methods

HHS has 10 regional offices that collaborate with state, tribal, local, and territorial HDs to implement and support policies and programs.^27^ HHS Region 2—the focus of this case study— includes 5 jurisdictions: New York State, New York City, New Jersey, Puerto Rico, and US Virgin Islands. With a combined population of nearly 33 million people living across 1,500 miles, these states and territories differ in population size, economics, demographics, and environments. They include racial, ethnic, and other underserved communities with distinct public health needs and assets, providing unique collaboration opportunities to address health equity barriers.^28^

We used a qualitative approach to better understand health equity strategies during the initial Omicron surge, from November 2021 to April 2022. The team first conducted a limited survey, followed by key informant interviews, to examine HD experiences in HHS Region 2 jurisdictions. The aim of the initial survey was to gather information to help develop the interview guide for the key informant interviews. In June 2022, we sent an email to state/territory epidemiologists to identify the person responsible for health equity to complete the survey. The email included a link to the survey. The anonymous online survey included 21 closed- and open-ended questions to assess successful interventions and barriers encountered (see Supplemental Materials, www.liebertpub.com/doi/suppl/10.1089/hs.2023.0001). The responses were received at the CDC via secure encrypted REDCap software.^29^ Five people completed the survey. Based on the responses and the thematically analyzed answers to the open-ended survey questions, we created a guide for the key informant interviews.

Key informant interviews were conducted using the semistructured interview guide (see Supplemental Materials), which provided in-depth insight into HDs' experiences addressing health inequities during the COVID-19 pandemic. Twelve health equity subject matter representatives from the 5 jurisdictions were purposefully sampled as key informants. From July 13 to August 1, 2022, we conducted 5 interviews (45 to 60 minutes each) with each jurisdiction using Microsoft Teams and facilitated by 1 lead interviewer and 3 notetakers. We obtained verbal consent from participants for notetaking during interviews. Interview questions focused on populations at increased risk for COVID-19, interventions, successes, gaps, and barriers. Key informant responses were transcribed into Microsoft Excel. HDs did not receive compensation for the interviews or survey. This activity was reviewed by CDC and was conducted consistent with applicable federal law and CDC policy.^30-34^

Following the key informant interviews, we analyzed the transcribed interviews to examine HD perspectives on impact, barriers, and gaps related to health equity interventions during the initial Omicron variant period. We conducted a thematic content analysis of data coded independently by 3 study team members; common themes were determined through consensus.^35-38^ Within common themes, we summarized associated topic areas and key lessons learned regarding health equity strategy implementation across jurisdictions.

## Results

**Figure 1.**
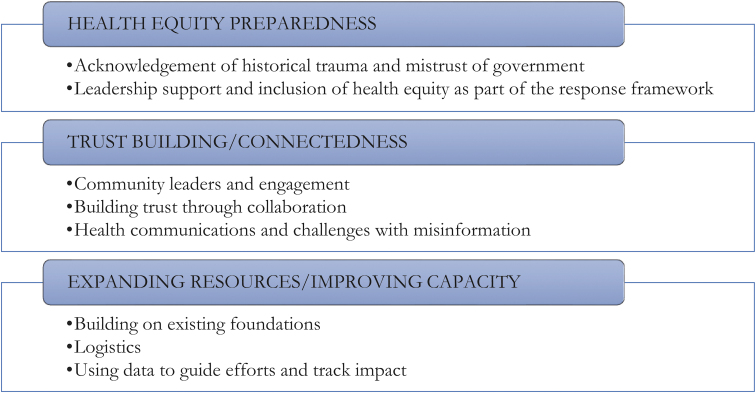
Common themes and associated topic areas.

Experiences and insights gathered from the surveys and interviews with HHS Region 2 HDs are reported by theme. Common themes identified include (1) health equity preparedness, (2) trust building/connectedness, and (3) expanding resources/improving capacity, each of which is aligned with associated topic areas (Figure 1). The topic areas are highlighted by quotes from key informants.

### Health Equity Preparedness

#### Acknowledgment of Historical Trauma and Mistrust of Government

Acknowledgment of historical experiences and harm was an important initial step in addressing health inequities. “Culturally, there is a history of treating citizens as guinea pigs. There is a different political relation between [our government], that cannot be erased or ignored.” Another key informant shared that their HD made acknowledgment of harm done as part of the health equity plan, including asking the local government to take accountability for health inequities:

*First was acknowledging the hurt […] we wanted to acknowledge harm done and we made this a part of the health equity plan. [Our] agency leaned on [the government leader] to make apologies, as well as the agency and partners to have accountability.*

Another stated:

*Medical investigations or studies were done […] with different populations and that is something that you can see a lot in social media. For this reason, persons express concern over pharmaceutical experimentation.*

#### Leadership Support and Inclusion of Health Equity as Part of the Response Framework

HDs found it essential for government leadership and policymakers to prioritize health equity as a vital part of the response framework from the beginning. This helped to lay the groundwork for health equity efforts and allowed better use of resources across agencies. One representative reported that the establishment of an emergency act by their jurisdiction's government leadership allowed HDs to work quickly to begin addressing health inequities. Another stated, “[Our] vaccine equity task force was developed at beginning of COVID-19 [… our health department leadership] prioritized health equity and included it in all policy and programming.”

Leadership of health equity efforts varied by jurisdiction, sometimes involving 1 individual and other times a group of people. A representative described the appointment of an equity officer as part of the centralized institutional structure, which allowed the HD to direct resources. In another jurisdiction, ongoing work with government leadership led to the establishment of a health equity working group that included influential and trusted people in their respective communities. One person described a task force comprising “senior staff of color” who were delegated to address inequities in their jurisdiction. Throughout the pandemic, this task force coordinated activities and leveraged senior-level partnerships across local agencies.

Several representatives stated that health equity was a focal point for vaccination planning. One specifically stated that their leadership emphasized that “Equity is number 1. [And that] this vision allowed for a lot of people to get vaccinated, especially people of color.” One interviewee described a minority health team established before the COVID-19 pandemic, which provided the groundwork for the development of a task force. Other efforts included the creation of a new health equity division overseeing COVID-19 vaccine administration and a supplemental grant program that funded mobile vaccinations; nursing; and educational interventions for people experiencing homelessness, with a mental illness, and with limited mobility.

### Trust Building/Connectedness

#### Community Leaders and Engagement

HDs found that working with trusted community leaders to deliver public health messages mitigated distrust. To conduct community outreach, HDs engaged with partners and leaders from faith-based organizations, community-based organizations, entertainment industries, and private businesses. Key informants indicated higher uptake of community interventions when collaborating with trusted community members. One key informant stated:

*Because of our community's small size, our relationships were longstanding. We had formed preexisting relationships prior to COVID-19. We engaged with federally qualified health centers, physicians, and faith-based organizations. Most of our employees were a part of some faith-based organizations so we leaned on those relationships.*

Previously established partnerships enabled jurisdictions to expeditiously identify and engage populations that were disproportionately affected by COVID-19. One HD worked with partners to streamline the identification of people who had disabilities or were homebound. Another representative stated that the knowledge of their jurisdiction's community and housing occupancy allowed for interventions to focus on individuals' routine interactions with healthcare services. Others said:

*The [health department] was provided with a list of names and contact information from municipalities for people who were homebound, as well as leaders and people who have experience serving this [population] group.*

*We reached out to housing authorities and found those that may be hard to reach. We made flyers beforehand to have hard copy handouts to share with people about what we were doing.*

*[We] relied on partners for [persons experiencing homelessness], when COVID hit. [We] had to build an internal state team, then connect with partners that run shelters. They would tell us what was needed… We worked with social services to ensure people [experiencing homelessness] were connected to COVID hotels […] We leaned heavily on partners to guide us, and as conduits for resources—staffing, vaccines, food […] partners shared needs and implementation.*

Culturally responsive outreach, in which HDs activated linguistically and culturally representative outreach teams, was indicated among the most successful interventions. One representative described “trainings for community-based leaders. It includes a collective of Black/Brown providers and physicians because the message is important, but so was the messenger.” Another action was hosting in-person community conversations where culturally appropriate messaging and vaccine testimonials were shared with attendees.

Reaching migrant populations was especially challenging. Due to a change in migration patterns during COVID-19, persons transitioning between countries or states could be lost to follow-up. Furthermore, people with undocumented status were fearful of participating in vaccinations or testing services. “The undocumented community [was] afraid to get vaccinated,” a representative explained, and “[that their] information would be shared with immigration.” Outreach methods were tailored to reach migrants, requiring a lot of groundwork as well as the inclusion of immigrants in the outreach planning. One interviewee stated:

*We were able to educate and communicate to undocumented populations that we would not collect sensitive information and the government would not be after them following vaccination and or testing. […] We did a lot of awareness building that [vaccination clinics were] a safe place to come.*

#### Building Trust Through Collaboration

Community- and faith-based populations in HHS Region 2 had different responses to COVID-19. Some populations were supportive of public health measures from the beginning, some were resistant, and others were open to collaboration if implemented in a setting of trust and mutual respect. Representatives spoke of low vaccination rates in certain communities, with public perceptions of COVID-19 vaccines and testing services sometimes impacted by people's religious and cultural beliefs. One person described a “disbelief in prevention measures and non-support of [prevention measures by] religious leaders in these communities,” and another spoke of segments of the population who were “resistant and hesitant due to religious beliefs.”

HHS Region 2 public health officials recognized the value that faith-based partners can bring in a response. “We needed to work intimately with communities of color,” said one representative. “The faith based are the trusted source, if [they] say ‘show up,’ the community shows up—[this is] effective with those that don't trust the news.” Another remarked, “We held town halls with [religious leaders]. We knew if we didn't get leaders involved, vaccination would be an uphill battle.”

Collaborations with faith-based leaders were described as instrumental in grassroots outreach, culturally appropriate messaging, and the facilitation of town hall meetings. In places where there may not have been previous collaboration between public health and faith-based organizations, new connections were established. Meetings with providers and timely communications were “often led with faith-based communities […] The faith-based leaders speak to community members, and their instruction is followed by people with noted hesitancy. We have built strong relationships with faith-based leaders.” Representatives commented about “how powerful these spiritual leaders are,” a “need to maintain relationship with [religious] leaders— dialogue and relationship to prepare to be engaged,” and an important lesson that “faith-based communities have to remain at the forefront.” Some stated:

*It took a long time to get some of these church figures on board. There was a disbelief in the prevention measures. It was very challenging…we have to get them to leave out politics and prioritize health of community. Sometimes [we have to invest in dialogue] for people to start listening, and once they do—[we] have to act.*

*It goes back to who is trusted in communities. The Black church has a long history in the community, it has space and the ability to invite people into spaces. We had resources—so it was a natural match—building on a wheel that people had already; once we had faith-based leaders on board.*

*Churches early on were not considered frontline establishments, but relationships were developed with the clergy members to get communications out.*

*Some religious institutions expressed that they did not agree with vaccinations, but we have been able to develop some alliances and partnerships with churches and religious institutions; it's made our entrance into that space much easier.*

#### Health Communications and Challenges With Misinformation

Misinformation about COVID-19 was a challenge. “We were competing with a lot of misinformation on social media that was big on anti-vaccination,” said a representative. “Anti-vaccination advocates were always ahead of us, and information was always changing.” Older adults were initially the most responsive to public health recommendations, but later in the pandemic, some began to question the need for vaccination. They equated COVID-19 to a “flu,” which they perceived to be less serious. This was most difficult during the initial Omicron variant period. Lastly, the public began questioning why they should get tested for COVID-19 when they assumed they already knew their diagnosis.

HDs used several strategies to counter the misinformation, including increased public health messaging, funding, and resources to community partners. “We made social media [using] TikTok and other platforms,” said a representative. “We incorporated 120-character text messaging to residences to try and beat the online misinformation.” Messaging and Facebook live chats were delivered in multiple languages. HDs also took steps to share statistics in plain language and communicated via billboards, television, and radio to address hesitancies based on vaccine myths. One person described the development of a program in which people would be able to speak with a licensed [health] professional at an event about any questions or concerns. “We had culturally competent persons regularly interact with these persons and educate them on COVID-19 and vaccines.” Other key informants stated:

*[Opinions of populations disproportionately affected by COVID-19] is important, particularly related to vaccine rollout. We established an information monitoring group, worked with organizations to monitor social media communications, particularly in English and Spanish on vaccine hesitation, opposition [… We] worked with organizations to counter misinformation—[we provided organizations] with language to counter.*

*The quick access to misinformation impacted pregnant individuals. Maternal mortality is discussed often [on social media]. There is a certain desperation people have [when they perceive] that there is not enough information on whether receiving vaccinations are safe.*

*To better serve the deaf community […] mobile vaccinations were organized in partnership with schools that have teachers who could communicate in sign language.*

HDs developed COVID-19 websites, hotlines, guidance, and appointment scheduling assistance in English or Spanish. HDs prioritized improving accessibility to COVID-19 information for people without reliable technology, limited English proficiency, and low literacy. Some jurisdictions recognized a need to develop materials in different languages, accessible formats, and at appropriate reading levels.

### Expanding Resources/Improving Capacity

#### Building on Existing Foundations

Having partnerships, resources, and infrastructure in place before the pandemic facilitated improving equity in COVID-19 response activities. One representative observed, “Before COVID, a goal was already determined to minimize inequity,” while another commented, “Building a relationship [with communities] started prior to COVID-19.” Another said:

*So much work was done prior to 2020 [with the health department examining] how to become an antiracist organization. [The health department had an] equity officer in the system prior to COVID. Operationally, this allowed us to direct resources.*

*We used a similar framework that was incorporated during the HIV response […] the plan was to mirror the HIV response.*

Jurisdictions also built upon experiences promoting health equity during other public health responses. One representative described lessons learned from a recent 2019 measles outbreak that focused on outreach strategies and grassroots community engagement with attention to “LGBTQIA+, adults 65+, Black people, Brown people, and certain religious communities.”

Longstanding community relationships provided HDs insight that informed resource needs, community partnerships, and development of outreach strategies that would work best for specific populations. “[We did] community focused reaching out—[using] sound system [loudspeakers] on cars, church in towns,” and in other places, “going to the country areas and reaching out through the churches was most effective.”

#### Logistics

While underlying causes of inequities could be complex and multifactorial, HDs reported that some challenges were logistical in nature. HD efforts to increase access to healthcare and support services were considered vital to promoting health equity among certain populations. “[We were] able to have teams employed, boots on the ground, out in field talking to individuals […] all [team] members were linguistically and culturally competent representatives, able to use soft skills, language, and [knowledge of local] culture as gateways to community to build trust.” One representative indicated that individuals who are homebound and people with disabilities experienced transportation barriers due in part to a dependency on others to access healthcare services. In 1 jurisdiction, the HD established clinics within 5 miles of disproportionately impacted zip codes and expanded community outreach. One interviewee reported that “rural [residents] do not have access […] we had to address this community need and how to get [people to these services].”

Mobile vaccination units were also seen as an important strategy to improve accessibility. As mass vaccination sites were reduced, 1 HD used pop-up sites with local culturally representative doctors at smaller venues such as town halls, shopping centers, and housing complexes. An interviewee observed that at-home vaccinations were the HD's most successful intervention among adults aged 65 years and older, people with disabilities, and homebound individuals.

#### Using Data to Guide Efforts and Track Impact

The use of data to promote health equity presented both challenges and opportunities. “[The health department] had to establish a centralized hub to look at and slice data by zip code and demographics,” a representative noted. “Each zip code would create data packets, which allowed us to mobilize and operationalize what we needed to do.” Another representative described the use of social vulnerability indicators to identify neighborhoods with the greatest inequity. One jurisdictional representative explained how they used zip code and geotracking data to identify areas of need based on lower numbers of people with health insurance. In another jurisdiction, the local health commissioner made data available to a steering committee with 7 workgroups assigned to populations at increased risk of COVID-19. Others stated:

*[We] started to think of methodology of how to identify risk factors that increased transmissions— occupation, overcrowding, multigenerational homes—stratified list of neighborhoods with greatest inequities.*

*We developed SAT scans [a software program that analyzes geospatial surveillance data] to identify clusters to direct testing and outreach with a hyperlocal approach. [We were able to] identify gathering spaces in neighborhoods [churches, mosques, parks, and other organizations], collaborating with different community members who have familiarity and relationships within communities.*

*I was a part of the team working with high-risk populations. We looked at policy as we looked at data. It was crosscutting and cross-sectional. We presented 3 times a week on processes and procedures for implementation. We built a vaccine plan with health equity embedded. This impacted a lot of people of color. It was one of the fastest fast-tracked programs.*

Key informants viewed the success of interventions by the number of people reporting awareness of testing and vaccination options, demonstrated the use of testing and vaccination services, and increased community champions utilizing culturally responsive resources. HDs tracked the impact of interventions by multiple means, including (1) weekly at-home vaccination data shared by providers, (2) daily vaccine perception and interaction data, (3) weekly program and clinical intervention effectiveness evaluation data, (4) data collection and analysis of disparities among people disproportionately affected by COVID-19 led by a community partners taskforce to ensure investment strategies into communities were current, and (5) immunization and epidemiological databases.

Key informants noted that gaps in data made it difficult to track inequities. One interviewee indicated challenges in collecting data to track vaccination status in the community, as well as data sharing among collaborators. “I believe [a major challenge is] not knowing how many we have reached. There are gaps in data.” Another interviewee said, “We have incomplete data on people who are homebound, in long-term care facilities, and people experiencing homelessness. Our greatest challenge is knowing how to determine this gap.”

### Lessons Learned and Remaining Challenges

Much was accomplished in HHS Region 2 to promote health equity. As a key informant stated:

*The next—testing phase—was more equitable, there was more collaboration, and it was less reactive. [During] the vaccine [phase] we got to really see the equity we built … but also got to see disparity. [We] had better data, more collaborators who were honest. The journey of equity from little to none to what it is now was remarkable.*

However, there were remaining gaps and challenges going forward. “Collectively as a nation, we need to take the lessons learned,” a representative noted. “There is a perception of things being done unsatisfactorily.” Protective factors identified included residing in communities where government leadership prioritized and invested in strategies to address health inequities before the pandemic, and in communities with strong social support networks that worked in partnership with local HDs. A couple of representatives stated:

*The government as an institution needs to be doing more community work, not just sitting at a desk. It is tiring on us to do administrative tasks than go and serve in the community. We need staff dedicated to community work. Our most successful strategy was with mobile vaccinations. We as the Department of Health should strengthen, expand, and maintain mobile services.*

*We have been able to develop partnerships with churches and religious leaders, [to] make entrance into space easier—leadership in church welcomes us, opens doors to institutions, [which] do play a role in vaccination rates and interest of people getting vaccinated.*

Box. Key Lessons Learned

- COVID-19 highlighted health inequities for certain populations and the need for jurisdictions to act to reduce those inequities.
- It is important to address misconceptions, misinformation, distrust, and concerns early to reach populations at increased risk.
- Leaning on existing relationships and creating new partnerships with community-based organizations, religious leaders, healthcare providers, and other key opinion leaders enabled more effective support to communities during the COVID-19 pandemic.
- Delivery of COVID-19 updates through community-trusted mainstream media channels reinforced key elements of public health messaging: familiarity, trust, and accountability.
- Needs and receptiveness varied for different populations, highlighting the importance of tailoring response efforts and resources, language accessibility, and inclusion of trusted representative messengers.
- Data gaps made it challenging to know precisely how well populations disproportionately affected by COVID-19 were reached.

One important lesson was to initiate health equity activities before a response rather than developing them reactively. “At the beginning, it was incredibly hard to build the equity model during the ‘emergency mode’ where the response was more reactive.” It is vital to integrate the lessons learned during COVID-19 “into routine operations, support recovery, and prepare… for future emergencies.” This requires a focus on “social determinants of health concerns, such as housing, food insecurity, access to healthcare.” Listening to communities and empowering them to develop solutions to address inequities in the social determinants of health should be prioritized. “Addressing social determinants of health,” a representative observed, “will lead to positive, sustainable change for individuals and communities at large.” One person suggested that jurisdictions may want to look for additional opportunities to engage with communities. Similarly stated, it is important to maintain relationships and trust that have been established (Box).

## Discussion

The COVID-19 response highlighted current and historical health inequities in the United States. By the second year of active response, public health officials, healthcare providers, first responders, and the nation were exhausted and suffering from COVID-19 fatigue, which was further complicated by the emergence of Omicron as a variant of concern. The Omicron variant accounted for the majority of cases in jurisdictions in the months to follow and added challenges to ending the COVID-19 pandemic during a time when restrictions were being lifted in the United States.^39^

Our case study findings indicate that community-driven approaches based on trust and existing partner relationships helped to effectively address many of the cultural barriers to the uptake of COVID-19 interventions by populations that have been disproportionately affected by COVID-19. Public health professionals in HHS Region 2 leveraged strong community partnerships to protect communities from the public health threat. Community outreach engaged collaborators from various sectors such as government, faith-based, and healthcare sectors to effectively address health inequities. Local community partners were positioned to provide essential services and support to people with functional and access needs.^40,41^ HD engagement of people representing impacted communities creates the capacity for flexible and diverse strategies to meet the needs of populations disproportionately affected by COVID-19. Having community-informed insights is imperative to the acceptance of public health interventions. It is important for HDs to address misinformation early in a response through clear messaging from trusted people. Improving the acceptability of interventions involves communications in plain language, messaging that emphasizes science over misinformation, engagement and publicizing by diverse trusted local leaders, and increasing accessibility to services.^42,43^

We acknowledge some limitations. State and local HD in other regions also implemented interventions to improve health outcomes among people disproportionately affected by COVID-19 in communities historically underserved by government programs and healthcare systems.^26^ However, the experiences described here may not be representative of racial and ethnic populations or communities in different US regions. Perspectives from HD representatives may be susceptible to social desirability bias. In addition, the key informants had to rely on their memory over several months to answer questions. We did not capture the nuanced perspectives of community partners, leaders, and practitioners who collaborated with the jurisdictional HDs.

There were noted gaps in public health data management systems (prior to an emergency). Areas for improvement include prioritizing and integrating health equity data to better inform interventions for populations disproportionately affected by COVID-19. We encourage continued efforts to identify and reach populations disproportionately affected by COVID-19 and improved strategies to better serve community needs. Furthermore, it is essential to take steps to understand protective factors, as well as the intersectionality, social, and structural context of populations disproportionately affected by COVID-19 that are underlying factors driving health inequities, which can compound disadvantages. Addressing the needs of diverse populations involves informed decisionmaking, diversity of thought, and delivery that are tailored to the community.

## Conclusion

Our findings may impact decisionmaking by government officials, public health professionals, community leaders, and healthcare systems in promoting health equity in current public health initiatives and during future public health responses. It is imperative to have an equity-centered approach to reduce and eliminate inequities in disease outcomes as individuals and communities continue to be impacted by the long-term effects of the COVID-19 pandemic.

## Supplementary Material

Supplemental data
