# Supplementary material for: New York State, New York City, New Jersey, Puerto Rico, and the US Virgin Islands' Health Department Experiences Promoting Health Equity During the Initial COVID-19 Omicron Variant Period, 2021-2022
Source: Health Secur. 2023 Sep 27;21(Suppl 1):S25–34. doi: 10.1089/hs.2023.0001 (PMC10818041; doi:10.1089/hs.2023.0001)
Supplement: Supplemental data [file SupplementalMaterial.zip › 23-0001 R1 Cox CS S1 RP Supplemental Material - Semistructured Interview Guide.docx]

**Supplemental Material 2. Interview Guide Questions**

| ***Overarching*** |
| --- |
| How would you describe the implementation of health equity in your jurisdiction during COVID-19? |
| ***Community of Interest*** |
| How did the health department identify specific populations to conduct outreach? |
| *What were the first actions the jurisdiction took to reach or assist [Identified] these groups?* |
| How have these groups been engaged throughout the pandemic? |
| How was engagement different during the Omicron surge? |
| What were the opinions of COVID-19 among these groups? |
| Can you explain your experience building trust with people in disproportionately affected communities? |
| ***Community Implementation Partnerships:*** |
| Thinking back to October 2021 until now **(pre-omicron)**, what was the public's perception of COVID-19 then and now? |
| Did religious, spiritual, or cultural practices have an effect on behaviors and following of health department recommendations? |
| Could you please share examples of why people had hesitancies or fears (for example: vaccine hesitancies, fears of government or pharmaceutical companies)? |
| Please provide specific examples of barriers to care. |
| Were these actual or perceived barriers to care? |
| How were these barriers addressed? |
| Does the health department have an existing relationship with local partners that support the outreach? |
| Was there a health equity unit established at the department of health? |
| If yes, what are the main goals or objectives of the unit? |
| What was the most trusted media for messaging/communications, and why? |
| **Lessons Learned** |
| Thinking about the survey, was there anything that we didn’t ask that you would like to share? |
| Were there vulnerable communities that were not reached? |
| Thinking about Long COVID, what interventions or support are being provided to disproportionately affected populations? |
