# Supplementary material for: New York State, New York City, New Jersey, Puerto Rico, and the US Virgin Islands' Health Department Experiences Promoting Health Equity During the Initial COVID-19 Omicron Variant Period, 2021-2022
Source: Health Secur. 2023 Sep 27;21(Suppl 1):S25–34. doi: 10.1089/hs.2023.0001 (PMC10818041; doi:10.1089/hs.2023.0001)
Supplement: Supplemental data [file SupplementalMaterial.zip › 23-0001 R1 Cox CS S1 RP Supplemental Material - Survey Questions.docx]

**Supplemental Material 1. Health Equity Jurisdiction Survey Questions**

| Was your jurisdiction implementing any interventions to address health equity during the Omicron surge (November2021 - April 2022)? |
| --- |
| Which of the following interventions was your jurisdiction implementing during the Omicron surge (Nov. 2021 - April2022) to address health equity? (Select all that apply)   - At home vaccinations - At home tests - Vaccine incentives (cash-based, waivers, certificates, etc.) - Alternate transport to testing or vaccination site (Lyft/Uber, public bus/subway system, taxi) - Culturally competent outreach (multilingual heath communication, partnerships with local community-based organizations, cultural/heritage events, etc.) - Other_____________________________ |
| Were any of the following groups identified as being disproportionately impacted by COVID-19? Select all that apply.  (*SVI=social vulnerability index)   - People experiencing homelessness - Black/African American - Hispanic or Latino - People with disabilities - Religious minority groups - Populations with High SVI* - Immigrants or refugees - Older adults (65+) - People from LGBTQIA+ communities - Incarcerated persons - Other_____________________________ - No group identified |
| The success of an intervention involves many factors including uptake by populations of focus, broad accessibility, and more. Which of the interventions selected (above) was most successful at reaching these populations? (Select one)   - At home vaccinations - At home tests - Vaccine incentives (cash-based, waivers, certificates, etc.) - Alternate transport to testing or vaccination site (Lyft/Uber, public bus/subway system, taxi) - Culturally competent outreach (multilingual heath communication, partnerships with local community-based organizations, cultural/heritage events, etc.) - Other_____________________________ |
| Please explain which groups (if identified above) this intervention reached and why it was successful. |
| Does this intervention have high acceptance in disproportionately affected populations? |
| Why do you think it was/ was not accepted? |
| Did the health department identify any barriers in accessing this intervention among disproportionately affected populations? |
| Please explain which barriers, and how barriers were resolved (if applicable). |
| Is your health department tracking the impact of the most successful intervention on reducing COVID-19 health disparities? |
| How is the impact being tracked? |
| Does your health department collect health equity-related data (for example, social risk domains such as food insecurity, housing insecurity, access to transportation) for this intervention? |
| What type of data and how is it collected? |
| Please describe types of partners. |
| Please describe specific resources tailored to reach disproportionately affected populations. Include any requirements needed to adapt resources. |
| Thinking into Fall 2022, what health equity issue do you think will be most important to address? |
| Are there plans to implement case surveillance of Long Covid? |
| What are the long-term strategies to address Long Covid in disproportionately affected populations? |
